# 2020 Clinical Highlights in Rheumatology

**DOI:** 10.31138/mjr.31.4.421

**Published:** 2020-12-28

**Authors:** Spyros N. Nikas

**Affiliations:** Private Practice Rheumatologist, Ioannina, Greece

**Keywords:** Highlights, literature, papers, research

As the year draws to a close, we would like to share some of the most interesting papers in the field of Rheumatology published in 2020, providing useful messages for everyday clinical practice.

Rheumatoid arthritis (RA) is a chronic autoimmune inflammatory disease, where glucocorticoids, conventional synthetic (cs), biologic (b) or targeted synthetic (ts) disease-modifying anti-rheumatic drugs (DMARDs) are the main therapeutic options. An interesting view, concerning the safety of these pharmacological interventions, was presented by Alexandre Sepriano et al., based on a systematic literature review of observational studies comparing safety outcomes.^1^ According to this, there is no difference in the risk of serious infections between the various bDMARDs; however, there is an increased risk with bDMARDs compared to csDMARDs. Additionally, there is no difference in the risk of herpes zoster infection across bDMARDs, but there is a difference between some tsDMARDs and bDMARDs (eg, tofacitinib compared with abatacept, in favor to abatacept). The risk of cancer for bDMARDs is similar to csDMARDs, while an increased risk of lower intestinal perforation with tocilizumab was noted, compared with csDMARDs or TNFi. Finally, tsDMARDs may share a possible increased risk of venous thromboembolism.

Management of RA patients with a prior malignancy remains a challenge, since bDMARD administration in this specific group of patients is still a controversial issue. However, according to a recent metanalysis (12 studies, 13,598 patients - 32,473 patient-years of follow-up), it seems that there is no increased risk of developing new or recurrent cancer in RA patients exposed to bDMARDs compared to csDMARDs.^2^ Most of the studies concerned TNFi, where the relative risk of new or recurrent cancer vs. csDMARDs was 0.95 (95% CI = 0.83).

Management of RA patients with biologics during pregnancy is another major treatment challenge, however recent data seems to be reassuring. Nicole W Tsao et al., after performing a systematic review and meta-analysis of 24 observational studies, concluded that pregnant women with inflammatory systemic diseases, including RA, exposed to bDMARDs do not bear an increased risk for congenital anomalies (adjusted OR 1.18, 95% CI: 0.88, 1.57).^3^

Methotrexate (MTX) is considered the cornerstone of RA management; exposure of RA patients to MTX seems to be associated with significantly lower risk of developing type 2 diabetes compared to RA patients not exposed to it.^4^ This encouraging news for a condition associated with increased cardiovascular risk, is based on a systematic review and meta-analysis of 16 studies, where the risk of type 2 diabetes was found decreased in RA patients using methotrexate (relative risk 0.48, 95% CI 0.16, 1.43). On the other hand, cytopenias, considered to occur with MTX in any rheumatic disease, are not common, according to a recent review of RCTs.^5^ In particular, the incidence of anaemia seems to be around 2.55% (95% CI 0.60–5.47%), of leukopenia 1.17% (95% CI 0.16–2.80%), and of thrombocytopenia 0.19% (95% CI 0.00–0.86%).

Axial Spondyloarthritis (axial SpA) is a chronic inflammatory disease predominantly affecting the axial skeleton and in general not associated with detectable serum autoantibodies. However, Quaden D et al. presented data implying an autoimmune basis in disease pathogenesis, and therefore, new tools for early axSpA diagnosis, at least in a patient subset.^6^ Identification of antibodies to 3 of 9 novel UH-axSpA peptides (Hasselt University [UH]-axSpA peptides) were significantly more likely to be found in patients with early axSpA patients in 2 independent cohorts (14.2% [22/155]) compared to patients with non-specific chronic low back pain (5% [4/75]), resulting in a specificity of 95%.

The pharmacological management of axSpA includes NSAIDs and biologics (TNFi or anti-IL-17A). In patients with TNFi failure, according to the Swiss Clinical Quality Management cohort, initiation of anti-IL17A agent or an alternative TNFi is associated with comparable effectiveness, since no significant differences were found in BASDAI50 responses at 1 year.^7^

Pain relief, improvement of quality of life, and inhibition of radiographic progression are the main treatment goals in axSpA. However, TNFi administration does not seem to protect against spinal radiographic progression over a course of 2 years or, according to other studies, of ≥4 years, although a possible protective effect might be seen after ≥4 years of treatment.^8^ No differences were seen, either with NSAIDs or anti-IL17A inhibitors (secukinumab) compared to a control group, in a treatment course of up to 2 years.

The use of anti-IL-17A biologics is a relatively new treatment approach for psoriatic arthritis compared to the much better known TNFi, and it is interesting to know the differences in terms of efficacy and safety between anti-IL-17A and TNFi in biologic-naïve patients with active psoriatic arthritis. Two head-to-head trials published this year showed that secukinumab (EXCEED study) or ixekizumab (SPIRIT) were not superior to adalimumab, at least in terms of musculoskeletal manifestations.^9,10^ However, both anti-IL-17A regiments were more effective in skin manifestations, while secukinumab seemed to be associated with a higher treatment retention rate than adalimumab (treatment discontinuation by week 52 14% vs 24%).

For the subset of PsA patients under TNFi, co-administration of MTX does not seem to offer any benefit compared to TNFi-monotherapy, at least in terms of disease activity, based on DAS28. However, this strategy seems to be associated with longer biologic survival.^11^

Encouraging findings come from two recent clinical trials concerning inhibition of the interleukin-23 (IL-23)/T-helper 17 cell pathway, a possible novel biologic therapeutic agent. Guselkumab (monoclonal antibody that specifically inhibits IL-23 by binding the cytokine's p19 subunit) seems to be efficacious with an acceptable safety profile in PsA patients with active biologic-naive (DISCOVER-2) or biologic-resistant (DISCOVER-1) disease.^12,13^

Approved drugs for systemic lupus erythematosus management are few, with belimumab being the only currently licensed biologic agent. It is recommended as an add-on therapy for the treatment of adult patients with active, autoantibody-positive SLE with high degree of disease activity, mainly involving the skin or musculoskeletal system. It was quite interesting that recently, IV belimumab administration was expanded to patients with active lupus nephritis, added to standard therapy (mycophenolate mofetil or cyclophosphamide-azathioprine), resulting in more favourite renal response than patients randomized to placebo.^14^

Giant cell arteritis is one the most inflammatory diseases in Rheumatology, where the diagnosis might be tricky in patients without cranial features. Van der Geest et al., after a systematic literature review, revealed that the most characteristic clinical finding indicative of the disease is limb claudication, providing a positive likelihood ratio (LR) of 6.01 (95% CI, 1.38–26.16), following by jaw claudication (positive LR, 4.90; 95% CI, 3.74–6.41), temporal artery thickening (positive LR, 4.70; 95% CI, 2.65–8.33), and temporal artery loss of pulse (positive LR, 3.25; 95% CI, 2.49–4.23).^15^ Among laboratory tests, platelet count of greater than 400 × 103/μL or erythrocyte sedimentation rate greater than 100 mm/h may also contribute to disease diagnosis. In another study, the significance of ultrasound compared to MRI as a first line imaging modality in GCA diagnosis is highlighted.^16^ Glucocorticoids still are the cornerstone of treatment; however, it seems that almost half of patients (47.2% [95% confidence interval 40.0, 54.3]) might experience disease relapse, mainly those with short duration of glucocorticoid administration.^17^

Biologics are used in the majority of rheumatic diseases, and osteoporosis is not an exception. Denosumab is considered one of the most effective and safe treatment approaches; however, as a biologic agent, is characterised by a class effect safety profile, consistent with higher incidence of serious adverse events of infections (RR, 1.21; 95% CI, 1.04–1.40; I2 = 0%), mainly of ear, nose, or throat.^18^ Of interest, no increased risk of any infection (RR, 1.03; 95% CI, 0.99–1.06) or infection-related mortality was found.

Fibromyalgia is another challenging rheumatic disease, characterised by chronic widespread musculoskeletal pain. Disease management includes both medication and self-care strategies, like exercise. Even though there is high-quality evidence in favour of cognitive behavioural therapy or central nervous system depressants or antidepressants as treatment approaches for quality of life or pain, unfortunately, efficacy appears to be small, not exceeding the minimum clinically important change.^19^

Knee osteoarthritis is a common but difficult to handle degenerative condition, since most management options confer moderate clinical benefit. Physical therapy or glucocorticoid intra-articular injections are included among the strongly recommended options,^20^ and according to findings of a recent clinical trial, the first approach seems to be superior to the second.^21^ In particular, at 1 year of follow up, patients undergoing physical therapy seem to experience less pain and functional disability than patients randomised to injection.
